# La dysplasie fibreuse: état des lieux

**DOI:** 10.11604/pamj.2015.21.21.3487

**Published:** 2015-05-08

**Authors:** Nessrine Akasbi, Fatima Ezzahra Abourazzak, Sofia Talbi, Latifa Tahiri, Taoufik Harzy

**Affiliations:** 1 Service de Rhumatologie, CHU Hassan II, Université Sidi Mohammed Ben Abdellah, Fès, Maroc

**Keywords:** Dysplasie fibreuse, diagnostic, bisphosphonates, perspectives thérapeutiques, fibrous dysplasia, diagnosis, bisphosphonates, therapeutic perspectives

## Abstract

La dysplasie fibreuse des os est une affection osseuse bénigne congénitale mais non héréditaire, où l'os normal est remplacé par un tissu fibreux renfermant une ostéogenèse immature. Elle est due à une mutation du gène GNAS 1sur le chromosome 20q13, une mutation activatrice de la sous-unité α de la protéine G. C'est une pathologie qui est le plus souvent silencieuse, de découverte fortuite sur une radiographie standard ou révélée par une douleur osseuse ou une fracture pathologique. L'imagerie et l'histologie, quand elle est nécessaire, permettent d’établir le diagnostic. Bien qu'il ne s'agisse pas d'une tumeur, elle est souvent classée dans la catégorie des tumeurs osseuses bénignes pour des raisons de diagnostic différentiel radiographique et anatomopathologique. Elle peut être monostotique ou polyostotique. L'approche thérapeutique est essentiellement symptomatique. Quelques publications récentes ont suggéré l'intérêt majeur d'un bisphosphonate, en particulier le pamidronate, qui diminuerait les douleurs et stimulerait une reminéralisation progressive des zones ostéolytiques chez les patients traités. D'autres traitements tels que la thérapie ciblée sont en cours d’évaluation.

## Introduction

La dysplasie fibreuse est une maladie osseuse congénitale bénigne rare. Il s'agit d'une prolifération focale de tissu fibreux au sein de la moelle osseuse due à une anomalie de différentiation des ostéoblastes. Cette maladie entraine des lésions ostéolytiques, des douleurs osseuses, des déformations, des fractures par fragilisation osseuse et parfois des complications neurologiques par compression nerveuse [1, 2]. C'est une pathologie qui est due à une mutation activatrice du gène GNAS 1 (guanine nucleotide binding protein, alpha stimulating) touchant les cellules somatiques [3]. Elle représente 2,5% des maladies osseuses et 7% des tumeurs osseuses [4]. La dysplasie fibreuse peut être monostotique ou polyostotique ou fait partie du syndrome de McCune-Albright. L'avènement des bisphosphonates dans le traitement de la dysplasie fibreuse a bouleversé l’évolution et le pronostic de cette maladie.

## Seminaire

### Etiopathogénie

La dysplasie fibreuse est une maladie congénitale due à une mutation du gène GNAS 1. C'est une mutation activatrice de la sous-unité α de la protéine G, impliquant une stimulation de l'adénylate cyclase, et une surproduction d'AMPc puis une surexpression de la protéine c-fos qui est à l'origine d'un défaut de différentiation des ostéoblastes. Cette mutation affecte d'autres types cellulaires que les précurseurs ostéoblastiques, notamment les mélanocytes de la peau et les cellules de nombreuses glandes endocrines (gonades, thyroïde, hypophyse, surrénales’) [5, 6]. Les lésions osseuses contiennent de nombreux préostéoblastes, mal différenciés, avec une matrice anormale, mal et irrégulièrement minéralisée, ces cellules sécrètent des cytokines qui activent localement la différenciation et l'activation des ostéoclastes, responsables de l'expansion de la lésion lytique dans les espaces trabéculaires et des érosions au niveau des corticales adjacentes [7]. Les cellules ostéoblastiques mal différentiées produisent en excès le receptor activator of nuclear factor kappa-B ligand (RANKL) qui se fixera sur son récepteur le RANK situé sur le précurseur ostéoclastique impliquant la voie de signalisation nuclear factor-kappa B (NFKB), responsable d´une maturation de cellules ostéoclastiques, et donc une hyper résorption osseuse [8]. Elles produisent aussi un excès d'interleukine 6 (IL-6), une cytokine pro inflammatoire qui induit une hyperactivité ostéoclastique, entraînant des lésions ostéolytiques au sein du tissu fibreux ainsi que dans l'os sain environnant [9]. Les cellules osseuses dysplasiques secrètent la protéine Fibroblast Growth Factor 23 (FGF-23) qui favorisent une fuite rénale de phosphore ce qui provoque un rachitisme ou une ostéomalacie vitaminorésistante, souvent associé à cette maladie [10, 11]. Le diabète phosphaté doit être systématiquement dépisté, car il est présent chez au moins la moitié des sujets atteints de dysplasie fibreuse polyostotique.

### Epidémiologie

La prévalence de la dysplasie fibreuse est inconnue, on peut considérer qu'elle est de 1/30000. Elle représente 2,5% des maladies osseuses et 7% des tumeurs osseuses. La dysplasie fibreuse des os touche également les deux sexes, le sexe ratio est de 1. L’âge au diagnostic est le plus souvent compris entre 5 et 30 ans [3]. Les lésions apparaissent généralement dans l'enfance et peuvent progresser avec la croissance squelettique. Le plus souvent, les lésions osseuses évoluent très peu après la puberté. Le risque de transformation sarcomateuse est de 0.5 à 4% selon les séries [4]. Tous les os peuvent être touchés. L'atteinte peut être monostotique ou polyostotique. Dans la forme monostotique qui représente 70 à 80% des cas, les atteintes les plus fréquentes sont les côtes (45%), l'extrémité céphalique et le col du fémur, l'os maxillaire, la voûte du crâne. Aux os longs, l'atteinte est typiquement métaphyso diaphysaire. Dans la forme polyostotique, il s'agit souvent d'une distribution unilatérale ou à prédominance unilatérale des sites osseux atteints [12]. Cette forme peut être associée à des manifestations cutanées et endocriniennes entrant dans le cadre du syndrome de Mac Cune-Albright.

### Quand poser le diagnostic de la dysplasie fibreuse?

Le diagnostic de la DF se base sur un faisceau d'arguments cliniques, biologiques et surtout radiologiques, et peut être confirmé par l’étude anatomopathologique.

### Signes cliniques

La dysplasie fibreuse des os est souvent asymptomatique, de découverte fortuite sur une radiographie standard faite pour une autre raison. Elle peut être révélée par une douleur qui peut être d'origine osseuse ou articulaire, lorsqu'une arthrose secondaire est présente. La douleur résulte souvent d'une fissure, ou d'une fracture pathologique ou d'une déformation osseuse avec handicap fonctionnel. Les déformations au cours de la dysplasie fibreuse sont de type de crosse de berger du tibia, du fémur ou de l'humérus, un genu varum ou un genu valgum, et une inégalité de longueur des membres inférieurs [13]. L'atteinte de la base du crane peut se manifester par une asymétrie faciale, une exophtalmie, des anomalies du développement de l'articulé dentaire.

### Formes particulières

Le syndrome de McCune-Albright associe une puberté précoce, une DF polyostotique, des taches cutanées café-au-lait et des anomalies endocriniennes à type de nodules thyroïdiens avec hyperthyroïdie, une hyperplasie surrénale avec hypercorticisme, des tumeurs hypophysaires avec acromégalie ou hyperprolactinémie [14]. Le syndrome de Mazabraud associe une DF et des myxomes intramusculaires qui se situent en général au voisinage des lésions osseuses, et ont tendance à récidiver après exérèse chirurgicale [15].

### Apport du bilan biologique

Les marqueurs de remodelage osseux sont utilisés pour évaluer l'activité de la maladie et la réponse au traitement. On note une élévation des marqueurs de formation osseuse: les phosphatases alcalines totales (PAL), les phosphatases alcalines osseuses (PAO) et l'ostéocalcine et ceux de la résorption osseuse: l'hydroxyprolinurie, les produits de dégradation du C-télopeptide du collagène de type I (CTX). Les marqueurs classiques de la DF sont la PAL qui est élevée chez environ 75% des patients, mais surtout la PAL osseuse et l'hydroxyprolinurie dont le taux est corrélé à l'extension et à la sévérité de la maladie. Les autres marqueurs peuvent aussi augmenter dans la DF [16, 17]. Le diabète phosphaté doit être dépisté. La phosphorémie est basse mais parfois normale. Il est calculé par la mesure de la réabsorption rénale du phosphore (TmPi/GFR), le dosage de FGF-23 sanguin n'est pas recommandé. La carence en vitamine D est fréquente chez les patients atteints de DF, puisque 25% de ces patients atteint de DF ont une valeur de 25-OHD sérique inférieure à 20 ng/ml avec une hyperparathyroïdie secondaire, d'où l'intérêt de son dosage systématique [18].

### Apport de l'imagerie dans le diagnostic de la dysplasie fibreuse

**Figure 1:**
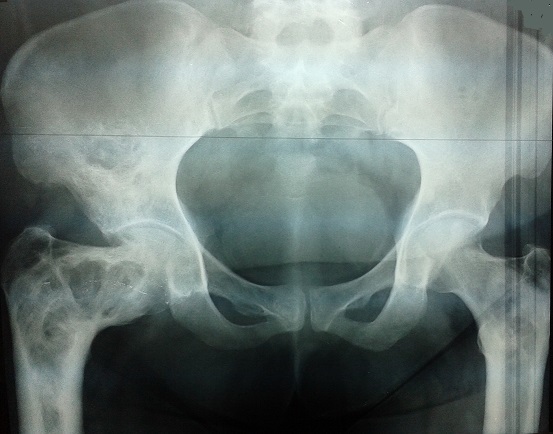
Lésion de dysplasie fibreuse de l'extrémité supérieure du fémur bilatérale accentuée à gauche

Le diagnostic est confirmé le plus souvent par les constatations radiologiques. Celles-ci vont mettre en évidence une lésion ostéolytique, souvent hétérogène, principalement radio-transparente, avec une corticale amincie, parfois une hypertrophie osseuse, avec souvent un liseré d'ostéocondensation périphérique à la lésion, mais avec à certains endroits une condensation osseuse dite « en verre dépoli » très évocatrice du diagnostic [19]. Les sites de prédilection sont la diaphyse des os longs, les métaphyses, en particulier le col fémoral, les côtes, le rachis et la région crânio-faciale. La TDM permet d’évaluer le risque de fracture, en mesurant l’épaisseur des corticales, et repérer des fissures invisibles sur la radiographie standard. La scintigraphie osseuse est pratiquée en début de la prise en charge pour dépister l'ensemble des lésions osseuses de la DF, les lésions dysplasiques sont hyperfixantes. L'IRM n'a pas d'intérêt dans le diagnostic de DF, mais si suspicion de transformation maligne [4] (Figure 1).

### Signes histologiques

L’étude anatomopathologique, quand elle est nécessaire, met en évidence une prolifération focale fibreuse au sein du tissu osseux, faite de cellules préostéoblastiques produisant de façon anarchique une matrice osseuse immature. La biopsie peut s'avérer très utile dans toutes les situations où le contexte clinique et les examens d'imagerie n'ont pas permis d’établir le diagnostic. La recherche de la mutation du gène GNAS 1, sur un tissu osseux ou endocrinien est possible [4]. Il a été montré par analyse histomorphométrique réalisée sur des biopsies osseuses en tissu lésionnel et en tissu non lésionnel [4] qu'il existait des lésions franches d'hyperparathyroïdie secondaire en tissu dysplasique, dont la présence et l'importance étaient corrélées à la concentration sérique en PTH [10].

### Diagnostic positif: HAS 2012 [20]

Sous l’égide de la Société Française de Rhumatologie (SFR), de plusieurs sociétés savantes, des centres de référence de “maladies rares”. Un travail sur la dysplasie fibreuse a été conduit selon la méthode des ‘Recommandations pour la pratique clinique’ de la Haute Autorité de santé (HAS) en 2012, permettant d’établir des recommandations dans le diagnostic et la prise en charge de la DF. En générale, le diagnostic de la DF doit être évoqué devant les signes suivants: la présence de douleurs osseuses ou articulaires, ceci incite à faire demander des radiographies. Une distribution unilatérale (métamérique ou hémimélique) des sites atteints est évocatrice; des taches café au lait, des zones de pigmentation cutanée brunâtre, de grande taille et à bords déchiquetés; une puberté précoce: l'association « puberté précoce-dysplasie osseuse polyostotique-taches café au lait » permet d'affirmer le diagnostic de syndrome de McCune-Albright; une déformation avec hypertrophie, le plus souvent asymétrique, de la voûte crânienne ou du massif facial; la découverte fortuite d'une lésion radiographique, ostéolytique ou condensante, soufflante, d'allure tumorale.

### La prise en charge thérapeutique de la dysplasie fibreuse

#### Buts

L'objectif principal est de contrôler l'activité de la maladie, de réduire la douleur, de prévenir les complications de la maladie, et d'améliorer la qualité de vie des patients.

#### Avènement des bisphosphonates

L'existence des lésions osseuses lytiques, et la présence d'une hyper résorption osseuse ont conduit à essayer les bisphosphonates dans le traitement de la DF, cette indication hors autorisation de mise au marché (AMM). Les indications des bisphosphonates dans la dysplasie fibreuse selon les recommandations de la HAS 2012 [20] sont: douleurs osseuses, rebelles au traitement symptomatique habituel, antalgique ou sous AINS; des lésions fragilisantes à risque de fracture sans indication chirurgicale et associées à une élévation des marqueurs du remodelage osseux; l'efficacité sera évaluée par l'utilisation d'une échelle de la douleur (EVA), des marqueurs osseux, et de l'imagerie radiographique.

#### Pamidronate (Aredia)

Le pamidronate est le produit le plus utilisé dans le traitement de la DF. Il est prescrit à raison de 90 mg, pendant 3 jours de suite soit un total de 180 mg tous les 6 mois, pendant 2 à 3 ans, il sera prescrit, par la suite, en fonction de la gravité et de la réponse au traitement. Dans la littérature, on dispose des données d'une série de 58 patients atteints de DF traités pendant une durée moyenne de quatre ans [18]. Cette étude a montré une réduction de l'intensité des douleurs en réponse au traitement par pamidronate, avec une disparition complète de la douleur osseuse chez 60% des patients, 24% de réponses partielles et seul patient non répondeur. Il s'est avéré que la proportion des répondeurs s'accroît avec le nombre des cures de pamidronate, par un effet additif de ces cures. L'aspect radiographique s'est amélioré nettement chez environ 50% des patients, sous forme d'un épaississement des corticales et d'un comblement des lésions ostéolytiques. Dans cette étude 12 patients qui avaient une atteinte de l'extrémité supérieure du fémur et qui ont bénéficié de mesures de la densité minérale osseuse (DMO) sur ce site, ont eu une augmentation significative de 15% de la DMO en site dysplasique. Cependant ce traitement ne s'accompagnait pas d'amélioration radiologique chez les enfants et les adolescents, malgré la réduction du remodelage osseux [21].

#### Alendronate

Dans la littérature, quelques cas de dysplasie fibreuse ont été traités par l'Alendronate. On décrit le cas d'un patient âgé de 79 ans ayant une dysplasie fibreuse des membres dans sa forme polyostotique depuis 55 ans, ce patient a été traité par Alendronate 5 mg/jour pendant 8.5 ans. L’évolution a été marquée par une amélioration de la douleur dès le huitième mois du traitement, avec une chute des phosphatases alcalines osseuses (PAL) au 24^ème^ mois du traitement, une amélioration de la densité minérale osseuse de 26,2%, en absence d'effets secondaires [22]. Trois autres patients ayant une dysplasie fibreuse de la base du crane responsable de céphalées intenses, ces patients ont été traités par une forte dose journalière d'Alendronate 40 mg/j pendant 6 mois. L’évolution a été marquée par la réduction significative des céphalées à 2 mois de traitement avec comme effet indésirable une ‘sophagite chez un patient. Les auteurs ont souligné le risque d'ostéonécrose mandibulaire à cette forte dose journalière de bisphosphonates [23]. Le cinquième cas, il s'agit d'une fille âgée de 10.5 ans ayant un syndrome de Mc cune albright avec une DF sévère et étendue. A l’âge de 7 ans, elle a reçu du Pamidronate: 1 mg/kg/j pendant 3 jours chaque 6 mois pendant 1 an (2 cures) avec une intolérance à la perfusion de pamidronate. Puis elle a eu une cure d'Alendronate 10 mg/jour pendant 30 jours entre les 2 cures de pamidronate sans efficacité. La décision était d'augmenter la dose de l'Alendronate 70 mg/semaine de façon continue pendant 1 an. L’évaluation de la douleur a passé de 8 à 5 sur l’échelle d’évaluation de la douleur (EVA) à 7 mois, puis a passé à 3 à 10 mois et puis a disparu totalement à 12 mois de traitement. On a noté une apparition de bandes de comblement osseux sur les métaphyses des poignets, une chute de l'hydroxyprolinurie, une normalisation des phosphatases alcalines, avec un effet de rémanence 1 an après l'arrêt du traitement [24]. On rappelle aussi le cas d'une patiente âgée de 22 ans traitée pour DF dans le cadre du syndrome de Mccune-albright par pamidronate 90 mg en perfusion chaque les 3 à 4 semaines pendant 3 mois et vue le récidive des douleurs, un traitement par Alendronate 10 mg/jour a été démarré, une amélioration spectaculaire de la densité minérale osseuse de 158% au niveau de sa hanche après 2 ans de traitement a été observé avec amélioration parallèle de la douleur osseuse et des marqueurs de remodelage osseux [25].

#### Risedronate

L'efficacité des bisphosphonates par voie orale ou voie veineuse dans le traitement de la DF à conduit à la réalisation d'un essai européen multicentrique appelé PROFIDYS pour tester un bisphosphonate orale qui est le Risedronate à la dose de 30 mg par voie orale, selon un schéma intermittent à raison d'un comprimé par jour pendant deux mois tous les six mois [19]. Ce médicament sera testé chez deux population, chez 78 patients ayant des lésions osseuses de DF et douleurs osseuses pour évaluer l'effet antalgique du Risedronate sur la DF (étude 1, durée de 1 an) et chez 78 patients ayant des lésions osseuses de DF sans douleur au début pour évaluer l’évolution radiologique sous traitement (étude 2, durée 3 ans).

#### L'acide zolédronique

L'acide zolédronique a été utilisé chez des patients atteints de DF par certaines équipes avec de bons résultats. On dispose, l'Aclasta 5 mg et le zometa 4 mg en perfusion unique répétée tous les six mois.. L'acide zolédronique peut avoir une efficacité similaire de celle du pamidronate et de l'alendronate, et peut être utilisé en première intension.

#### Protocole des bisphosphonates [20]

L'adaptation et la durée du traitement sont fonction du résultat obtenu. En cas de disparition complète des douleurs sous traitement au bout de 24 mois, un arrêt du traitement, une diminution de moitié de la dose ou un espacement du rythme des perfusions peuvent être envisagés; en cas de réapparition des douleurs, le rythme de perfusion peut être rapproché; en cas d'effondrement du taux des marqueurs du remodelage osseux (CTX), une interruption du traitement ou un espacement des cycles doit être discuté.

#### Traitements adjuvants

Tout traitement par bisphosphonate doit être associé à une évaluation et éventuelle correction d'une insuffisance ou carence en calcium et en vitamine D. Chez l'adulte les apports sont de 1g/jour de calcium et 800 UI/jour de vitamine D3. Chez les patients hypophosphatémiques, l'objectif thérapeutique est d'améliorer la phosphorémie. La dose habituelle est de 1 200 à 1 600 mg/j de phosphore. Dans le traitement de la DF, on prescrit des médicaments pour lutter contre la douleur. On dispose des antalgiques selon les paliers de l'OMS, des anti inflammatoires non stéroïdiens (AINS), des médicaments à visée anti neuropathique: amitriptyline, prégabaline… Parfois une dose de corticothérapie de 1 mg/kg/j pendant une courte durée est nécessaire si syndrome de compression nerveuse notamment la compression du nerf optique.

#### Le traitement chirurgical

Il s'impose en cas de fracture, ou en prévention de fracture, la chirurgie de décompression peut être aussi proposé.

### Perspectives thérapeutiques

#### Le dénosumab: anticorps anti RANKL

Les cellules dysplasiques produisent en excès le RANKL qui en se fixant sur son récepteur induit une hyper ostéoclastose et une hyper résorption osseuse [8]. En se basant sur ces données physiopathologiques, une équipe américaine [26] a testé le dénosumab chez un patient atteint de DF. Il s'agit d'un enfant de 9 ans qui présente une DF avec une lésion extensive du fémur depuis 4 ans avec échec de traitement par pamidronate pendant 1 an. La biopsie de la lésion a montré un aspect compatible avec une DF avec une surexpression du RANKL. On rappelle que le Dénosumab est anticorps anti RANKL, utilisé dans le traitement de l'ostéoporose post ménopausique et des métastases d'origine prostatique. Les auteurs ont testé le Dénosumab à la dose de 1 mg/kg/mois en augmentant de 0.25 mg/kg chaque les 3 mois pendant un an de traitement. L’évolution a été marquée par la réduction de la douleur, des marqueurs de résorption osseuse et de la masse de la DF.

#### Le tocilizumab

Le tocilizumab est un anticorps monoclonal humanisé qui bloque l'action des récepteurs de l'interleukine 6 largement utilisé dans le traitement de la polyarthrite rhumatoïde. Le rationnel de l'essaie du tocilizumab dans le traitement de la DF se base sur le fait que les cellules dysplasique secrètent en excès l'interleukine 6 qui contribue aux lésions ostéolytiques et à la déminéralisation osseuse au cours de la DF [9]. Une étude européenne randomisée en double aveugle contre placebo est en cours, testant le tocilizumab sur 12 patients, les résultats préliminaires sont très encourageants.

## Conclusion

La dysplasie fibreuse des os est une maladie osseuse bénigne rare d’étiopathogénie qui reste mal élucidée malgré certaines découvertes physiopathologiques intéressantes. Le traitement se base essentiellement sur les bisphosphonates qui ont bouleversé l’évolution et le pronostic de la maladie. D'autres traitements tels que la thérapie ciblée sont encore d’évaluation.
